# The Kallikrein-Kinin System: A Novel Mediator of IL-17-Driven Anti-*Candida* Immunity in the Kidney

**DOI:** 10.1371/journal.ppat.1005952

**Published:** 2016-11-04

**Authors:** Kritika Ramani, Abhishek V. Garg, Chetan V. Jawale, Heather R. Conti, Natasha Whibley, Edwin K. Jackson, Sruti S. Shiva, William Horne, Jay K. Kolls, Sarah L. Gaffen, Partha S. Biswas

**Affiliations:** 1 Department of Medicine, Division of Rheumatology and Clinical Immunology, University of Pittsburgh, Pittsburgh, Pennsylvania, United States of America; 2 Department of Biological Sciences, University of Toledo, Toledo, Ohio, United States of America; 3 Department of Pharmacology and Chemical Biology, University of Pittsburgh, Pittsburgh, Pennsylvania, United States of America; 4 Vascular Medicine Institute, Dept. of Medicine, University of Pittsburgh, Pittsburgh, Pennsylvania, United States of America; 5 Richard King Mellon Foundation Institute for Pediatric Research, Children's Hospital of Pittsburgh, Pittsburgh, Pennsylvania, United States of America; University of Rochester, UNITED STATES

## Abstract

The incidence of life-threatening disseminated *Candida albicans* infections is increasing in hospitalized patients, with fatalities as high as 60%. Death from disseminated candidiasis in a significant percentage of cases is due to fungal invasion of the kidney, leading to renal failure. Treatment of candidiasis is hampered by drug toxicity, the emergence of antifungal drug resistance and lack of vaccines against fungal pathogens. IL-17 is a key mediator of defense against candidiasis. The underlying mechanisms of IL-17-mediated renal immunity have so far been assumed to occur solely through the regulation of antimicrobial mechanisms, particularly activation of neutrophils. Here, we identify an unexpected role for IL-17 in inducing the Kallikrein (Klk)-Kinin System (KKS) in *C*. *albicans*-infected kidney, and we show that the KKS provides significant renal protection in candidiasis. Microarray data indicated that Klk1 was upregulated in infected kidney in an IL-17-dependent manner. Overexpression of Klk1 or treatment with bradykinin rescued IL-17RA^-/-^ mice from candidiasis. Therapeutic manipulation of IL-17-KKS pathways restored renal function and prolonged survival by preventing apoptosis of renal cells following *C*. *albicans* infection. Furthermore, combining a minimally effective dose of fluconazole with bradykinin markedly improved survival compared to either drug alone. These results indicate that IL-17 not only limits fungal growth in the kidney, but also prevents renal tissue damage and preserves kidney function during disseminated candidiasis through the KKS. Since drugs targeting the KKS are approved clinically, these findings offer potential avenues for the treatment of this fatal nosocomial infection.

## Author Summary

*Candida albicans* is the causative agent of oropharyngeal candidiasis (OPC, thrush), dermal and vaginal candidiasis. However, the most severe *C*. *albicans*-induced disease is disseminated candidiasis, a frequent nosocomial infection associated with a high mortality rate. During disseminated candidiasis, *C*. *albicans* form invasive hyphae that damage target organs, particularly kidney and liver. Previous studies have identified an essential role of interleukin-17 (IL-17) in controlling systemic infection through regulation of neutrophils. We show here for the first time that IL-17 also regulates the renal protective Kallikrein-kinin system (KKS). Our discovery of a connection between IL-17 and the KKS suggests a new, previously unanticipated avenue for the treatment of renal damage in disseminated candidiasis. These findings have potential translational significance**,** as agonists of the KKS are in routine clinical use. Therefore, these results not only identify downstream mediators that could serve as novel drug targets, but could possibly be used to guide decisions on whether targeting these mediators could be a useful therapeutic option in conjunction with current antifungal therapies.

## Introduction

The commensal fungus *Candida albicans* causes several clinical conditions in immunocompromised individuals, including oropharyngeal candidiasis (OPC, thrush) and vaginal candidiasis [1]. However, the most severe *Candida*-induced disease is a systemic form of bloodstream candidiasis. Disseminated candidiasis is the fourth most common hospital acquired infection and is associated with a 40–60% mortality rate [2,3]. Intravascular catheters, abdominal surgery, prolonged use of antibiotics and immunosuppressive therapy are risk factors for this disease, and contribute to the concerning rise in the incidence of candidiasis [1]. Available antifungal medications are limited by drug-drug interactions, drug resistance, toxicity and high treatment costs. To date, there are no effective vaccines to fungal pathogens [1]. Thus, there is an unmet clinical need to develop alternative, safe and ideally inexpensive approaches to treat this fatal infection.

Candidiasis is often treated effectively with azoles, amphotericin B and echinocandins [4,5]. The extensive and prolonged use of antifungal medications to treat systemic fungal infections, however, has led to drug resistant fungal strains and host toxicity [5,6]. Thus, novel antifungals or improved therapeutic strategies are still needed. Indeed, *in vitro* studies combining azoles with other drugs such as tacrolimus, cyclosporine A, amiodarone or retigeric acid B yielded encouraging results [7–11]. These data justify the concept of novel combination therapies to treat candidiasis at lower dosage in preclinical animal models.

Death due to sepsis is a frequent outcome of disseminated candidiasis [1]. However, in 30–40% adults and 50% neonates, *Candida* hyphae invade and injure the kidney, leading to irreversible damage and fatal renal failure [12]. Once *Candida* invades the kidney, a robust innate response dominated by neutrophils and monocytes/macrophages contributes to pathogen clearance and sets the stage for the adaptive immune response [13]. During the course of fungal clearance, both innate effector cells and kidney-resident cells release tissue repair enzymes and anti-inflammatory proteins. While necessary to repair injured tissue, these factors also limit bystander damage caused by innate immune cells.

Considerable data implicate IL-17 (IL-17A) in immunity to *C*. *albicans*. For example, IL-17RA^-/-^, IL-17RC^-/-^, RORγt^-/-^ and IL-17A^-/-^ mice are all susceptible to systemic *C*. *albicans* infection [14–17]. At mucosal surfaces, IL-17 mediates antifungal activity by driving the expression of antimicrobial peptides and chemokines that facilitate neutrophil influx [18,19]. Unlike mucocutaneous candidiasis, which affects individuals with compromised IL-17 signaling, systemic *C*. *albicans* infection normally impacts individuals with no known underlying genetic defects in IL-17 signaling pathways [1]. One recent study reported that IL-17 also acts on NK cells to drive the production of GM-CSF, with protective activities in disseminated candidiasis [15]. However, the mechanisms of local IL-17-mediated antifungal activities within the kidney still remain unclear.

**Fig 1:**
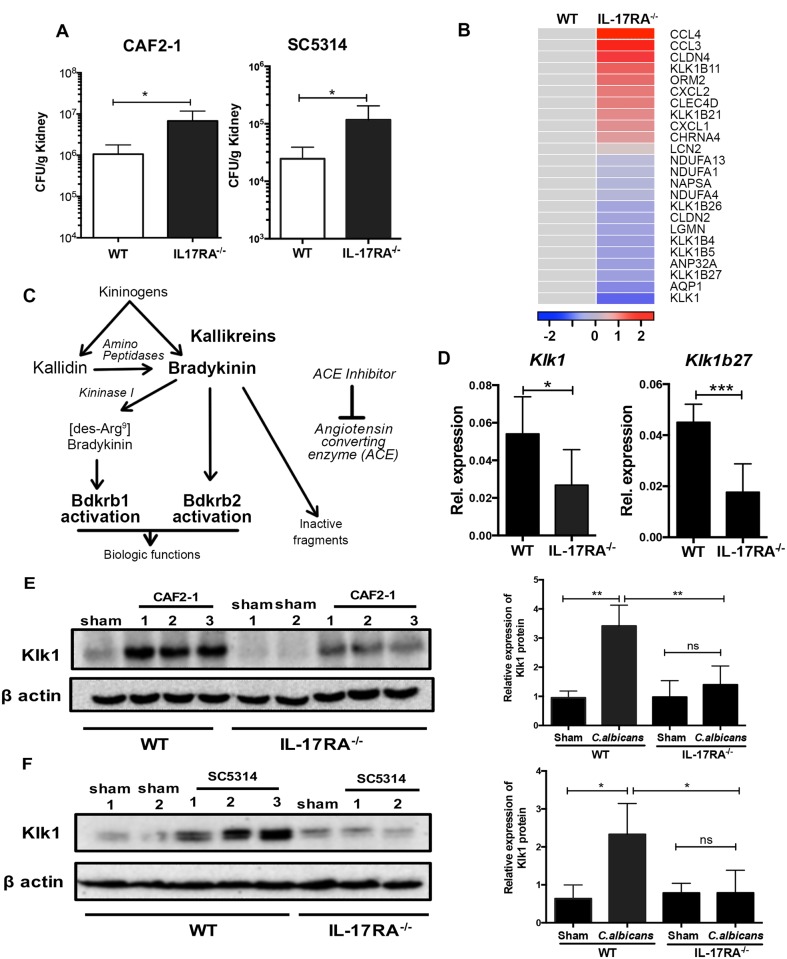
The expression of Klk is impaired in IL-17RA^-/-^ kidney following *C. albicans* infection. **(A)** WT and IL-17RA^-/-^ mice (n = 4–6) were subjected to systemic *C*. *albicans* (CAF2-1 or SC5314) infection. After 48 h, kidneys were evaluated for fungal load. Data pooled from 2–3 independent experiments. **(B)** Heat map representing averaged intensity of expression of genes in WT and IL-17RA^-/-^ kidneys (n = 2) at 48 h p.i. **(C)** Schematic representation of Kallikrein-kinin system (KKS). (**D)** Kidneys of WT and IL-17RA^-/-^ mice (n = 6) were evaluated for expression of *Klk1* and *Klk1b27* at 48 h p.i. Data pooled from 2 independent experiments. At 72 h p.i., whole cell extracts from WT and IL-17RA^-/-^ kidneys (n = 5–6) infected with *C*. *albicans* **(E)** CAF2-1 or **(F)** SC5314 were evaluated for Klk1 protein by western blotting. Sham infected mice received PBS. Images were quantified using ImageJ. Representative image of 1 of 2 independent experiments (**E** and **F**). For the bar diagram, data are combined from 2 independent experiments. Bars indicate mean ± S.D. *P*<0.05 (*), <0.01 (**), <0.001 (***). ns, not significant.

The Kallikreins (Klk) are a family of fifteen related serine proteases. Klk1 in particular plays a critical role in renal function and pathology [20]. Klk cleave kininogens to generate kinin peptides, known as bradykinin and kallidin. Collectively, this system is termed the Kallikrein-kinin system (KKS) (Fig 1C). Bradykinin signals through two receptors; bradykinin receptor β2 (Bdkrb2) is constitutively expressed, whereas Bdkrb1 is inducible upon inflammatory signals. The primary known function of the KKS is to regulate blood pressure by promoting vasodilation [20]. In addition, studies in animal models implicate the KKS in regulating inflammation, tissue repair and homeostasis during kidney injury [21]. The renal protective function of the KKS is mediated through upregulation of tissue repair proteins, inhibition of profibrotic factors, and control of apoptosis [21]. Consistently, polymorphisms in KKS-related genes (*ACE*, *BDKRB2*, *NOS3*, *KLK1)* are associated with an increased risk of acute and chronic renal injury in humans [22–25]. While it is clear that the KKS protects the kidney in disorders associated with sterile inflammation, its role in renal immunity in infectious settings is less well defined.

In some bacterial and viral infections, bradykinin enhances vascular permeability to facilitate pathogen spread [26,27]. However, there are very few studies linking the KKS and *C*. *albicans* pathogenesis. Kininogens have been shown to bind the *C*. *albicans* cell wall, causing the fungal SAP2 protease to induce release of biologically active kinins [28,29]. Additionally, the KKS was implicated in IL-17-mediated skin inflammation and an IL-17-dependent model of autoimmunity (experimental autoimmune encephaloymyelitis, EAE) [30,31].

Here, we identified kallikrein genes as novel IL-17 targets in disseminated candidiasis, revealing an unanticipated link between IL-17 and KKS-mediated renal protection. Mice lacking the IL-17 receptor A subunit (IL-17RA) exhibited diminished Klk expression in the kidney. Moreover, overexpression of Klk1 restored protective renal immunity against systemic *C*. *albicans* infection in IL-17RA^-/-^ mice. The IL-17-KKS-axis activated bradykinin receptors, which served to enhance renal anti-*C*. *albicans* immunity. Addition of exogenous bradykinin in immunocompetent mice prevented renal damage by inhibiting apoptosis of kidney-resident cells, and prolonged animal survival during candidiasis. Finally, addition of bradykinin to a minimally effective dose of fluconazole significantly improved survival. These data identify a previously unrecognized link between IL-17 and KKS-mediated renal protection against disseminated candidiasis, which may provide the basis for clinical intervention in this disease.

## Results

### IL-17 triggers Klk expression in the kidney during disseminated candidiasis

In *C*. *albicans* intravenous challenge in mice, the kidney is the most heavily colonized organ [32]. With a higher inoculum (>10^6^ cfu), mice succumb to infection within 48–72 h due to sepsis. However, mice infected with a low dose of *C*. *albicans* (10^5^ cfu) exhibit progressive loss of renal function over a period of ~2 weeks, which more accurately reflects disease progression in humans [32]. During candidiasis, IL-17 is rapidly upregulated in the kidney, but its function in that organ is unknown [14]. To understand how IL-17 mediates kidney-specific immunity, we performed Illumina microarray analyses comparing WT and IL-17RA^-/-^ renal gene expression at 48 h p.i. Confirming previous reports, IL-17RA^-/-^ mice demonstrated significantly increased kidney fungal burden in comparison to WT following infection with *C*. *albicans* (CAF2-1 or SC5314) (Fig 1A) [14,16,33].

The classic IL-17 gene signature includes neutrophil-related genes and antimicrobial peptides (AMPs), such as CXC chemokines (*Cxcl1*,*2*,*5*), defensins (*Defb3*), calprotectin (*S100a8*) and lipocalin 2 (*Lcn2*) [18]. Although a few genes previously shown to be controlled by IL-17 were differentially expressed in the kidney during candidiasis, overall we saw a surprisingly distinct gene profile compared to analyses of IL-17-dependent genes in other settings (Fig 1B and S1A Fig) [18]. The expression of classical IL-17-responsive genes such as *Cxcl1*, *Cxcl2* and *Lcn2* were unaffected in IL-17RA^-/-^ mice (S1A Fig). Using the DAVID Gene Functional Classification algorithm (which uses a gene-to-gene similarity matrix based shared functional annotation), we identified several functional groups with enrichment scores over 1.0. Most striking to us based on their known role in kidney physiology was the enrichment of genes encoding the KKS (Fig 1B and 1C). Multiple *Klk* genes were suppressed in the kidney of IL-17RA^-/-^ mice compared to WT following *C*. *albicans* infection. These results were verified by measuring the renal expression of *Klk1* and *Klk1b27* by qPCR (Fig 1D).

We next analyzed the impact of differential fungal load on *Klk* gene expression in the kidney by inoculating WT mice with either a high (10^6^ cfu) or low (10^5^ cfu) dose of *C*. *albicans* and measuring expression at 48 h p.i. Either dose caused comparable *Klk1* expression (S1B Fig), indicating that the differences in *Klk* expression are not due to differential fungal loads. We then verified protein expression of Klk1 by immunoblotting. Klk1 was constitutively expressed at comparable, albeit low, levels in the sham-infected WT and IL-17RA^-/-^ mice. However, Klk1 was upregulated in WT kidney following *C*. *albicans* infection. This observation was true upon infection with either the CAF2-1 or SC5314 strains of *C*. *albicans*. Confirming the gene expression data, we observed lower expression of renal Klk1 in the absence of IL-17RA during systemic infection (Fig 1E and 1F). Collectively, these data indicate that IL-17 signaling in the kidney regulates Klk1 expression during disseminated candidiasis. To our knowledge, this is the first demonstration that renal Klk1 is upregulated in an infectious setting, and certainly first demonstration that the KKS is controlled by IL-17.

### Klk1 is required for IL-17-mediated renal protection against disseminated candidiasis

**Fig 2:**
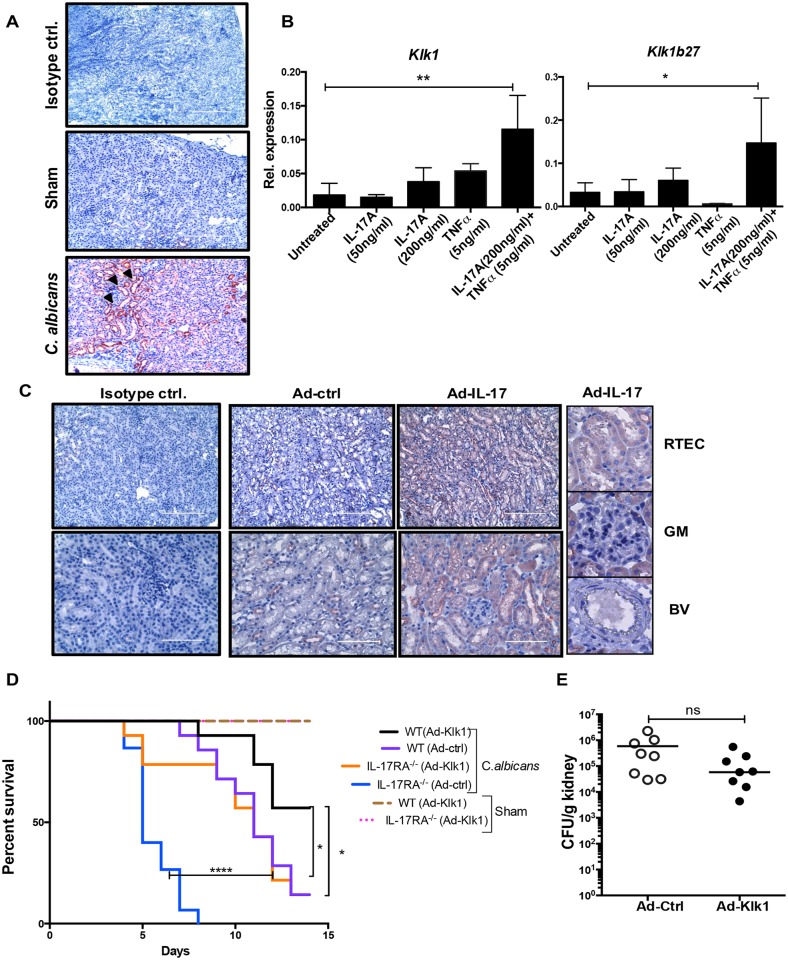
IL-17-driven Klk1 expression in the kidney is necessary for immunity to *C. albicans*. **(A)** WT mice (n = 5) were subjected to systemic candidiasis. Sham-infected mice received PBS. After 72 h, kidney sections were stained with anti-Klk1 or isotype control Abs. Black arrows indicate Klk1 staining. Photomicrographs are representative of 2 independent experiments. Original magnification: 100X. **(B)** Primary RTEC from C57Bl6/J mice were treated ± IL-17 (50 ng/ml and 200 ng/ml), TNFα (5 ng/ml) or IL-17 (200 ng/ml) + TNFα (5 ng/ml) for 24 h. Expression of *Klk1* and *Klk1b27* was assessed by qPCR. Bars represent mean ± S.D. Data are representative of 4 independent experiments. **(C)** WT mice (n = 5) were injected with Ad-IL-17 or Ad-ctrl (1x10^9^ pfu). Six days post-injection, kidney sections were stained with anti-Klk1 or isotype control Abs. Inset: Klk1 staining in RTEC and negative staining in glomerulus (GM) and blood vessels (BV). Photomicrographs are representative of 2 independent experiments. Original magnification: 200X (upper panel) and 400X (lower panel). **(D)** WT and IL-17RA^-/-^ (n = 14) mice were injected with Ad-Klk1 or Ad-ctrl 72 h prior to systemic *C*. *albicans* infection. Sham-infected WT and IL-17RA^-/-^ mice (n = 3) received Ad-Klk1 only. Survival was assessed over 14 d. Data pooled from 3 independent experiments. **(E)** WT (n = 8) mice were injected with Ad-Klk1 or Ad-ctrl 72 h prior to systemic *C*. *albicans* infection. Fungal burden was assessed at 72 h p.i. Each dot represents one mouse, and horizontal bars indicate mean. Data are combined from 2 independent experiments. *P* < 0.05 (*), <0.01 (**), <0.0001 (****). ns, not significant.

To understand the role of Kallikreins in candidiasis, we focused on Klk1 based on its connection to IL-17-driven diseases including EAE and systemic lupus erythematosus [30,31]. Kidney sections from *C*. *albicans*-infected WT mice were stained for Klk1 at 72 h p.i. Only kidney-resident cells, particularly renal tubular epithelial cells (RTEC), expressed Klk1 during infection (Fig 2A). These results agree with previous reports indicating that RTEC are the major producers of Klk1 in chronic kidney diseases [34,35]. Although Klk1 controls vital kidney functions, its regulation and function under inflammatory conditions are not well defined. We therefore asked whether changes in *Klk1* expression were a direct result of IL-17 signaling in RTEC, or a by-product of generalized renal inflammation due to increased fungal burden. We treated primary RTEC *in vitro* with IL-17 together with TNFα, a cytokine with which IL-17 exhibits strong signaling cooperativity [36]. Indeed, IL-17 and TNFα triggered strong synergistic upregulation of *Klk1* and *Klk1b27* mRNA (Fig 2B). Neither IL-17F nor IL-17C induced the expression of *Klk* genes (S1C Fig). Thus, IL-17 in conjunction with TNFα directly regulates *Klk* gene expression in RTEC, revealing a previously unrecognized class of IL-17-dependent target genes.

To verify the finding that IL-17 induces Klk1 in the kidney, we overexpressed IL-17 in WT mice using adenovirus (Ad-IL-17) [14]. Mice infected with Ad-IL-17 exhibited 400-fold more serum IL-17 than with a control vector (Ad-ctrl) (S2A Fig). This increased level of IL-17 was not associated with systemic inflammation, as serum TNFα and IL-1β levels were undetectable. By IHC, RTEC within kidney stained positively for Klk1 following overexpression of IL-17. The expression of Klk1 was restricted to RTEC, as no staining could be detected in the glomerular and vascular compartments of the kidney (Fig 2C). Additionally, Ad-IL-17 administration upregulated multiple IL-17 target genes in the kidney (*Il6*, *Cxcl5* and *Lcn2*) (Suppl. Fig 2B). Nonetheless, kidneys of Ad-IL-17-treated mice exhibited no overt inflammatory changes (S2C Fig). Thus, IL-17 induces the expression of Klk1 in RTEC following disseminated candidiasis.

Klk1 protects the kidney against acute and chronic disorders in sterile inflammation, but has not been linked to candidiasis or IL-17 signaling. To test the hypothesis that Klk1 plays a critical role in IL-17-driven renal protection against disseminated candidiasis, we overexpressed Klk1 with the adenoviral system (Ad-Klk1) and assessed disease susceptibility. Remarkably, overexpression of Klk1 significantly improved the survival of *C*. *albicans-*infected IL-17RA^-/-^ mice and in WT mice (Fig 2D). A previous study suggested that Klk1 may induce inflammatory cytokines in human RTECs, at least *in vitro* [37]. However, we found that overexpression of Klk1 had very little impact on renal inflammatory gene expression or fungal load (Fig 2E and S2D Fig). Overall, these data indicate that Klk1 enhances anti-*C*. *albicans* immunity in the kidney in an IL-17-dependent manner, but is not responsible for inducing inflammatory gene expression**.**

### Activation of the bradykinin receptors is required for IL-17-Klk1 axis-driven protection against disseminated candidiasis

**Fig 3:**
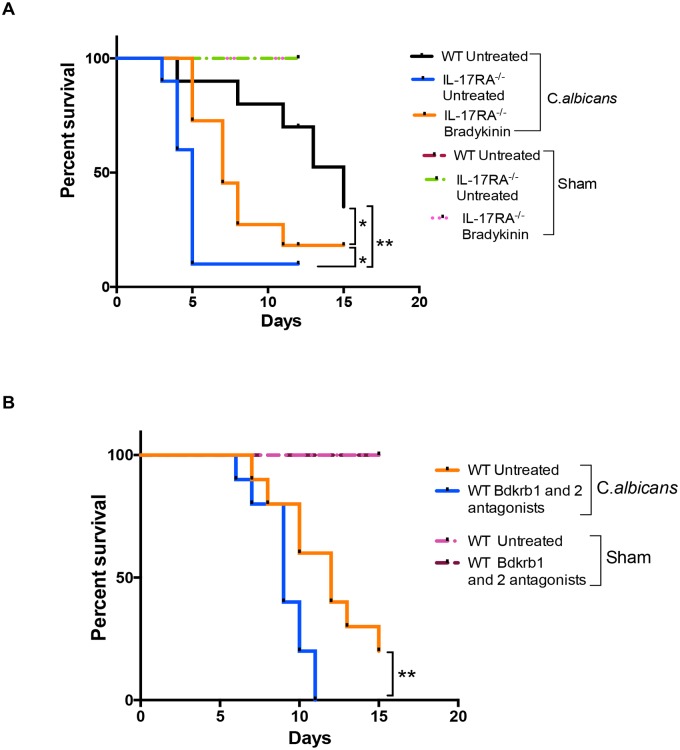
Bradykinin confers renal protection to IL-17RA^-/-^ mice following disseminated *C. albicans* infection. **(A)** IL-17RA^-/-^ mice (n = 14) were treated with bradykinin (300 nmol/kg/day) or PBS starting on day -1 (relative to infection). On day 0, mice were administered *C*. *albicans* i.v. and evaluated for survival over 14 d. WT mice were infected with *C*. *albicans* and left untreated. Sham-infected IL-17RA^-/-^ mice were given bradykinin only or left untreated (n = 3). **(B)** WT mice (n = 10–11) were treated with Bdkrb1 (R715; 1 mg/kg/day) and Bdkrb2 (HOE140; 1mg/kg/day) antagonists or PBS starting 1 day prior to infection and then daily for 14 d. Sham-infected WT mice were treated with the antagonists only (n = 3). Mice were evaluated for survival over 14 d. Data are pooled from 2 independent experiments for **(A)** and **(B)**. *P* <0.05 (*), <0.01 (**).

Klk1 mediates cleavage of kininogens to generate bradykinin, which signals through Bdkrb1 and Bdkrb2 (Fig 1C) [21]. Additionally, Klk1 activates protease-activated receptors (PAR) such as PAR4 to trigger the release of inflammatory mediators from RTEC [37]. To define the role of Bdkrb activation in renal immunity, IL-17RA^-/-^ mice were treated with bradykinin (300 nmol/kg) and survival evaluated following infection. As shown, 90% of the untreated IL-17RA^-/-^ mice succumbed to infection by day 5 p.i., while mortality in IL-17RA^-/-^ mice was delayed with bradykinin treatment (Fig 3A). Additionally, untreated WT mice had a modestly increased survival benefit compared to bradykinin treated IL-17RA^-/-^ mice, suggesting that there may also be a Bdkrb-independent pathway occurring in candidiasis.

Disseminated candidiasis typically impacts individuals with no known underlying immune defects [1]. Therefore, we assessed impact of Bdkrb signaling in mice with intact IL-17 signaling capacity and normal levels of Klk1. Accordingly, WT mice were treated with Bdkrb1 and Bdkrb2 antagonists and evaluated for survival following systemic *C*. *albicans* infection. Mice given Bdkrb1 and Bdkrb2 antagonists had significantly reduced survival compared to untreated controls (Fig 3B). Collectively, these results indicate participation of Bdkrb signaling in the renal host defense during disseminated candidiasis.

### Bradykinin prevents renal damage and preserves kidney function in immunocompetent mice following disseminated candidiasis

**Fig 4:**
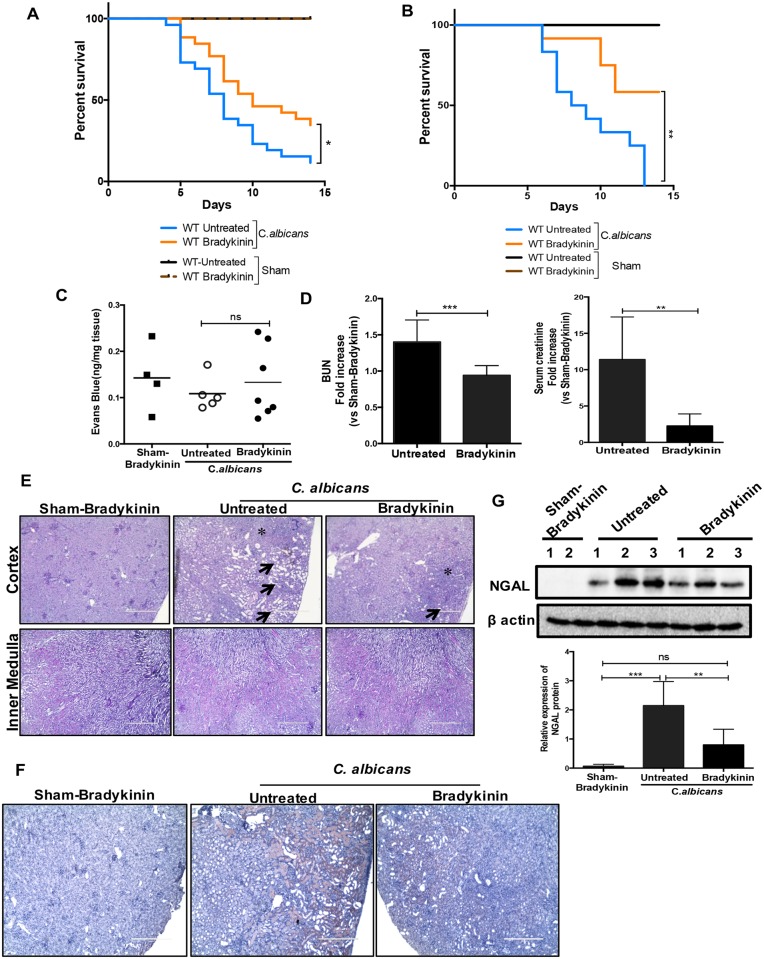
Bradykinin alleviated kidney injury and preserved renal function in *C. albicans* infected mice. WT mice (n = 14–20) were either left treated ± bradykinin (300 nmol/kg/day) starting day -1 (relative to infection). Mice were infected with *C*. *albicans* strains **(A)** (CAF2-1) or **(B)** (SC5314). Sham-infected WT mice were treated ± bradykinin (n = 3–5). Survival was assessed over 14 d. Data are pooled from **(A)** 4 and **(B)** 2 independent experiments. At day 7 p.i., mice were evaluated for **(C)** angioedema development in the hind paw (n = 4–7) and **(D)** serum BUN and creatinine levels (n = 6–10). Data pooled from 2–3 independent experiments. Each dot represents one mouse and the bars indicate mean. At day 7 p.i., kidney sections were evaluated for **(E)** histopathology and inflammatory cell influx by PAS staining (n = 8) and **(F)** NGAL expression by IHC (n = 6–8). Black arrows indicate tubular damage and atropy; * indicates inflammatory cell influx. Representative photomicrographs from 2 independent experiments. Original magnification: 100X. **(G)** Cell lysates of kidney homogenates (n = 5–6) were evaluated for NGAL by western blotting. Images were enumerated using ImageJ. Representative image of 1 of 2 independent experiments. Bars indicate mean ± S.D and combined from 2 independent experiments. P <0.05 (*), <0.01 (**). <0.001 (***), <0.0001 (****). ns, not significant.

Despite advances in antifungal therapy against disseminated candidiasis, mortality in patients with systemic *C*. *albicans* infection remains unacceptably high [1]. Since IL-17 is implicated in controlling candidiasis in experimental mouse models, targeting downstream mediators of IL-17 signaling pathway is an attractive approach to treat disseminated candidiasis [1]. In this regard, we hypothesized that manipulation of the IL-17-KKS pathway with bradykinin would ameliorate candidiasis in a host with intact IL-17 signaling. To provide proof-of-principle, WT mice were treated with bradykinin and survival assessed with two strains of *C*. *albicans* (CAF2-1 or SC5314*)*. Indeed, the bradykinin-treated cohort exhibited significantly delayed mortality compared to an untreated control group (Fig 4A and 4B). Although bradykinin has been implicated in the development of angioedema and hypotesion [38], mice treated with bradykinin did not show an increased incidence of angioedema at day 7 p.i. (Fig 4C). Moreover, to investigate the hypotensive effect of bradykinin, we performed a study where blood pressure (BP) alterations were measured in real time in uninfected WT mice following bradykinin treatment. We find that i.p. injection of bradykinin exerted only a very brief hypotensive response that peaked within ~1 minute with full recovery by 6 minutes (maximum reduction of Mean Arterial BP was 36%) (S3A Fig). This result indicates that bradykinin is not likely to have any long term consequences on renal function during candidiasis. Taken together, these results demonstrate a beneficial effect of exploiting the IL-17-KKS axis in treating disseminated candidiasis even in a host capable of mounting normal IL-17 response.

Following hyphal invasion, renal injury is mediated by unchecked fungal replication and bystander tissue damage caused by the local inflammatory response. Therefore, clearance of *C*. *albicans* and timely repair of damaged tissues is crucial to preserve renal function. To understand the mechanisms by which bradykinin mediates renal protection during disseminated candidiasis, WT mice were treated with bradykinin starting on day -1 and then daily for 7 days. Mice treated with bradykinin demonstrated significantly diminished serum blood urea nitrogen and creatinine levels compared to untreated animals (Fig 4D), indicating that bradykinin preserves normal renal function in systemic *C*. *albicans* infection.

We then asked whether improved renal function in bradykinin treated mice was due to reduced damage of kidney parenchyma following fungal invasion based on histological analyses. While sham-infected mice treated with bradykinin showed normal kidney histology, the renal parenchyma of infected mice showed overt pathological changes characterized by loss of brush border epithelium and tubular atrophy at day 7 p.i. Moreover, the damage was primarily restricted to the renal cortex and outer medullary region. In line with the kidney function results, renal damage was ameliorated upon bradykinin treatment (Fig 4E). Interestingly, the influx of inflammatory cells was comparable between the treated and untreated groups. Furthermore, we observed significantly diminished expression of neutrophil gelatinase-associated lipocalin (NGAL), a prototypical kidney injury marker, in bradykinin treated animals (Fig 4F and 4G). Collectively, these results indicate that bradykinin prevents *C*. *albicans*-mediated renal damage and preserves renal function during disseminated candidiasis.

### Bradykinin-mediated renal protection is independent of fungal clearance or inflammatory cell influx

**Fig 5:**
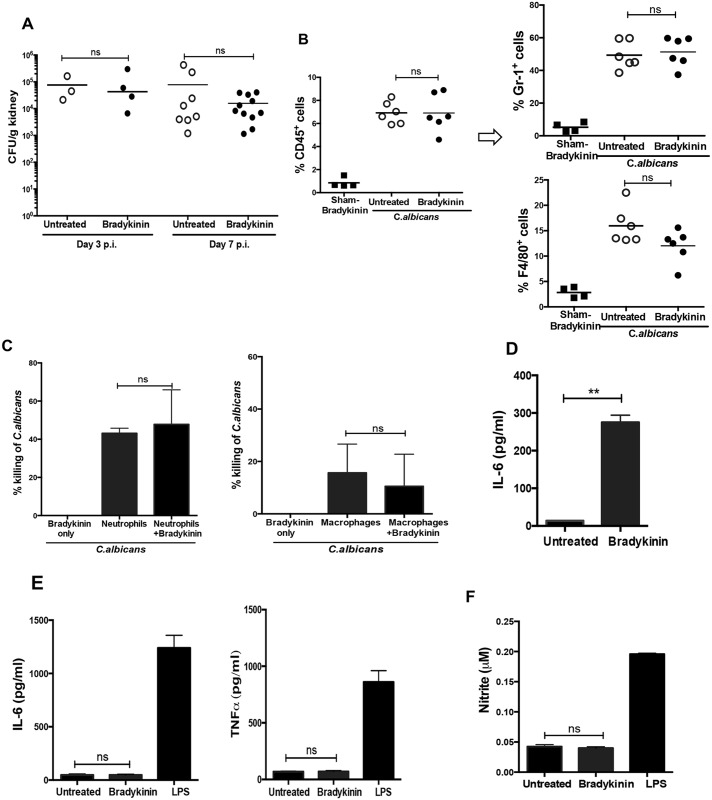
Minimal impact of bradykinin treatment on fungal clearance and inflammatory cells influx in the *C. albicans* infected kidney. WT mice were treated ± bradykinin (300 nmol/kg/day) or PBS on day -1 (relative to infection). **(A)** Kidneys were evaluated for fungal load on days 3 (n = 3–4) and 7 (n = 8–11) p.i. (**B**) At day 7 p.i. (n = 4–6), kidney infiltrating neutrophils (Gr1^+^) and macrophages (F4/80^+^) (gated on CD45^+^) cells were evaluated by flow cytometry. Data are combined from 2 independent experiments for **(A)** and **(B)**. Each dot represents one mouse, and the bars indicate mean. **(C)** BM-derived neutrophils and BMDMs from WT mice were incubated *in vitro* with unopsonized *C*. *albicans* yeast ± bradykinin for 3 h, and fungal load was determined by plating culture supernatants. Percentage of *C*. *albicans* killed by neutrophils and macrophages is shown. **(D)** IL-6 in RTEC conditioned media was assessed after 24 h bradykinin treatment. **(E)** IL-6 and TNFα and **(F)** nitrite levels in the supernatants of BMDMs treated ± bradykinin or LPS (1 ng/ml) for 24 h. Data are representative of 3 independent experiments for **(C-F)**. Bars indicate mean ± S.D. p <0.01 (**). ns, not significant.

To identify the effector mechanisms by which bradykinin prevents renal insufficiency, we evaluated fungal load and inflammatory cell influx at days 3 and 7 p.i. Although differences in kidney function were already evident early as day 7 p.i. (Fig 4D), fungal loads were comparable between the bradykinin treated and untreated groups at these time points (Fig 5A). Previous studies have shown that neutrophils and monocytes/macrophages mediate fungal clearance in candidiasis [39,40]. Thus, we examined the frequency of infiltrating myeloid cells in kidney upon bradykinin treatment. In agreement with the fungal clearance rates, the percentages of kidney infiltrating inflammatory cells (CD45^+^), neutrophils (Gr1^+^) and macrophages (F4/80^+^) were similar between groups (Fig 5B and S3B Fig). We next assessed whether bradykinin impacted the candidacidal activity of innate cells. Bradykinin treatment of BM-derived neutrophils and macrophages (BMDM) did not alter their ability to kill unopsonized *C*. *albicans* yeasts, as determined by an *in vitro* fungal killing assay (Fig 5C). Additionally, RTEC and BMDMs were stimulated *in vitro* with bradykinin and culture supernatants evaluated for cytokines and nitrite production. Bradykinin induced IL-6 in RTECs (Fig 5D), but did not induce IL-6, TNFα or nitrite in BMDMs (Fig 5E and 5F). Overall, these results suggest that renal protection observed in bradykinin treated mice is not due to diminished fungal load, inflammatory cell infiltration or proinflammatory function of innate cells in the kidney.

### Bradykinin prevents apoptosis of kidney-resident cells during disseminated candidiasis

Activation of bradykinin receptors protects the kidney from end-stage renal damage by inducing tissue-protective growth factors and matrix-degrading enzymes [21]. Therefore, we examined the effect of bradykinin treatment on profibrotic changes and tissue-protective growth factors expression in candidiasis. We observed minimal extracellular matrix protein deposition in bradykinin treated mice (S4A Fig). Additionally, there were no significant differences in expression of genes encoding tissue-protective growth factors (*Hgf and Ctgf*) or matrix-metalloproteinases at day 7 p.i. (*Mmp2* and *Mmp9*) (S4B Fig).

**Fig 6:**
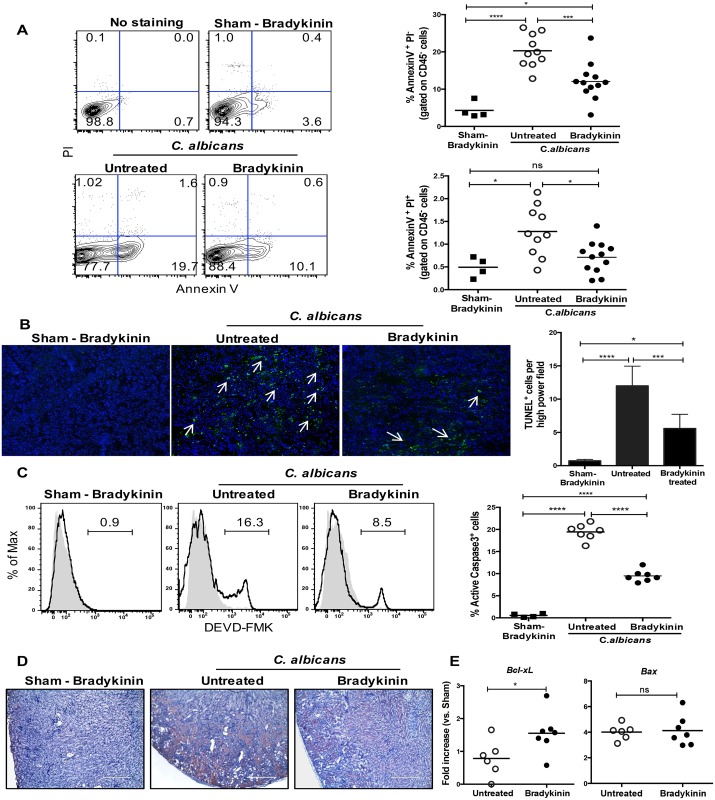
Apoptosis of kidney-resident cells is reduced in bradykinin treated mice in candidiasis. WT mice were treated with bradykinin (300 nmol/kg/day**)** starting 1 day prior to infection and daily for 7 days. Sham-infected WT mice were treated with bradykinin. **(A)** At day 7 p.i. (n = 4–12), early and late apoptotic kidney resident cells (gated on CD45^-^ cells) were quantified by AnnexinV and PI staining. **(B)** Frozen kidney sections (n = 4–8) were subjected to TUNEL staining and counterstained with DAPI. White arrows indicate TUNEL^+^ cells. The number of TUNEL^+^ cells was quantified in 15 randomly selected high powered fields (400X). Original magnification: 100X. **(C)** Frequency of cleaved Caspase-3^+^ kidney-resident cells (gated on CD45^-^ cells) were quantified by flow cytometry (n = 4–6). Solid histogram: Negative control; Open histogram: DEVD-FMK staining. **(D)** Serial kidney sections (n = 4–6) were stained for cleaved Caspase-3. (**E**) Sorted kidney-resident cells (CD45^-^) (n = 6–7) were evaluated for the expression of *Bcl-xL* and *Bax* mRNA by qPCR. Data pooled from 2 and 3 independent experiments for (**A-D**) and **(E)**, respectively. Each dot represents one mouse and bars indicate mean. Bar indicates mean ± S.D. *P* <0.05 (*), <0.01 (**), <0.0001 (****). ns, not significant.

Previous studies have described an anti-apoptotic function of bradykinin in kidney injury [41,42]. Therefore, we assessed apoptosis in kidney-resident cells following *C*. *albicans* infection. At day 7 p.i., flow cytometry analysis revealed a significantly reduced frequency of both early (AnnexinV^+^PI^-^) and late (AnnexinV^+^PI^+^) apoptotic kidney-resident cells (CD45^-^) in bradykinin treated compared to untreated mice (Fig 6A). Concurrently, there was a significant reduction in the number of TUNEL positive cells in the renal cortex following bradykinin treatment (Fig 6B). *C*. *albicans* can regulate survival of kidney-resident macrophages via Caspase-3 [39]. In agreement with the reduced apoptosis (Fig 6A), bradykinin treatment resulted in a significantly diminished number of cleaved Caspase-3^+^ kidney-resident cells (CD45^-^) (Fig 6C). Moreover, IHC indicated that cleaved Caspase-3 is localized in the renal cortex and outer medullary region of untreated mice (Fig 6D). The extent of cleaved Caspase-3 was markedly reduced following bradykinin treatment (Fig 6D). Although expression of *Bax* (a pro-apoptotic gene) in kidney-resident cells was comparable between the groups, there was an increase in expression of *Bcl-xL* (an anti-apoptotic gene) after bradykinin treatment (Fig 6E). Thus, bradykinin preserves renal function during systemic fungal infection by limiting apoptosis of kidney-resident cells.

### A minimally effective dose of fluconazole combined with bradykinin improved survival in disseminated candidiasis

**Fig 7:**
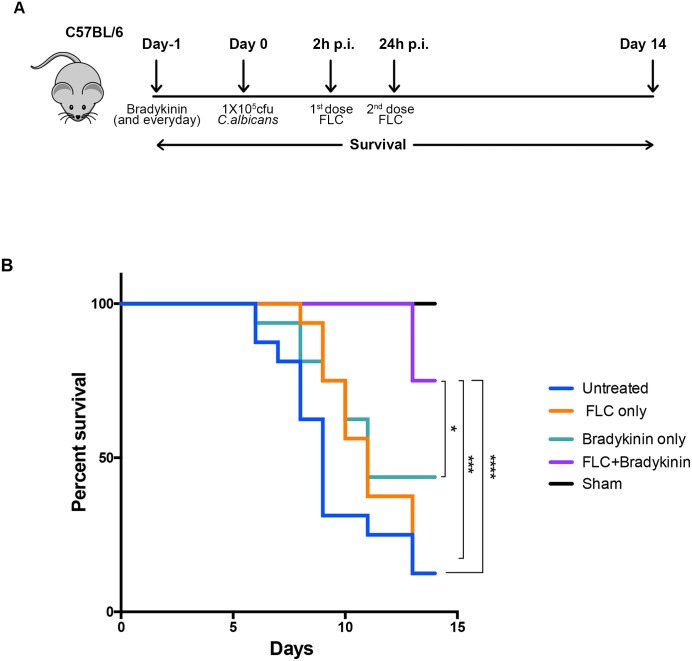
Combination of fluconazole and bradykinin increased survival of *C. albicans* infected mice. **(A)** Experimental design for combinatorial fluconazole and bradykinin therapy strategy **(B)** WT mice (n = 16) were infected with *C*. *albicans*. Infected mice were treated with two doses of FLC only (5 mg/kg at 2 and 24 h p.i.), bradykinin only (300nmol/kg starting 1 day prior to infection and daily for 14 days) or a combination of FLC and bradykinin. Sham-infected mice (n = 3) were treated with FLC, bradykinin or both. Survival was assessed over 14 d. Data are combined from 3 independent experiments. p<0.05 (*), p>0.001 (***), p>0.0001 (****).

There is an unmet clinical need to reduce the dosage of current antifungal drugs to overcome the problem of drug resistance and toxicity. Based on the renal protective function of bradykinin in disseminated candidiasis (Figs 3A, 3B and 4A), we hypothesized that a minimally effective dose of fluconazole (FLC) combined with bradykinin would confer better protection against disseminated candidiasis than either agent alone. To test this hypothesis, we first determined a minimally effective dose of FLC on WT mice with disseminated candidiasis. The lowest dose of FLC (5 mg/kg) was the least effective in clearing the fungus at day 4 p.i. (S5A Fig). Fungal clearance correlated well with survival, as mice treated with FLC (5 mg/kg) showed the same susceptibility as untreated animals (S5B Fig). Therefore, the 5 mg/kg dose of FLC was chosen to evaluate the impact of the combination therapy. Next, *C*. *albicans* infected WT mice were treated with FLC and/or bradykinin and evaluated for survival over 14 d (Fig 7A). Strikingly, mice receiving the combination of bradykinin and FLC showed a significant increase in survival compared to untreated mice or mice given FLC or bradykinin alone (Fig 7B). As expected, mice treated with bradykinin alone but not FLC demonstrated increased survival compared to untreated mice (Fig 7B). These data show that a combination of bradykinin and FLC confers better protection against disseminated candidiasis than either drug individually. Consequently, use of bradykinin could potentially permit reducing the dose of antifungal drugs without compromising efficacy against fungal infection.

## Discussion

Kidneys in a healthy state are sterile. However, renal infections occur via hematogenous routes or from ascending spread from the bladder or urethra [43]. In recent years, considerable data have implicated IL-17 in immunity against disseminated candidiasis [14–16]. Nevertheless, it is unclear how IL-17 regulates immunity within the kidney, the most heavily colonized organ during blood-borne *C*. *albicans* infection. In the present report, we have identified renal-protective kallikreins as novel IL-17 target genes in systemic candidiasis, thereby revealing a new connection between IL-17 and KKS-mediated renal defense. IL-17 not only limits fungal growth in the kidney, but also prevents renal tissue damage and preserves kidney function during *C*. *albicans* invasion (S6 Fig). Consequently, therapeutic manipulation of the IL-17-KKS pathways protected mice from early mortality in disseminated candidiasis. Our data provide important and potentially translatable insights into the renal functions of IL-17 in the context of this fatal hospital-acquired infection.

Kidney-specific immune responses are mediated by both kidney-infiltrating immune effectors and kidney resident cells. Although IL-17RA is ubiquitously expressed, most documented IL-17R signaling occurs in non-hematopoietic cells, particularly cells of epithelial and mesenchymal origin [44,45]. A recent study also suggested a role for IL-17RA signaling in NK cell development in the context of disseminated candidiasis [15], although this report conflicts with a study showing that NK cells are redundant for antifungal defense in immunocompetent hosts [46]. Consistent with the latter finding, we and others have observed upregulation of transcripts encoding IL-17A and IL-17-responsive genes in the kidney [14]. We also showed that kidney resident cells express the IL-17R and are responsive to IL-17 [47]. Taken together, these results argue that there is a bona fide, kidney-specific role of IL-17 in immunity to candidiasis.

Unlike mucocutaneous candidiasis, disseminated infection typically occurs in individuals with no known defects in IL-17 signaling pathways. In line with these observations, we were intrigued by the surprisingly different gene profile seen in *C*. *albicans*-infected kidney compared to prior studies involving mucosal *Candida* infection such as the tongue [18]. These results highlight the fact that different cell types “interpret” IL-17 signals differently, with non-identical patterns of gene expression depending on setting. Therefore, lessons derived from studies of anti-*C*. *albicans* immunity at mucosal sites cannot always be applied to local kidney immune responses. Although we show that IL-17 (in conjunction with TNFα) induces *Klk1* gene in primary RTEC, there is little known about *Klk1* gene regulation at the transcriptional level. We have identified conserved CCAAT Enhancer Binding Protein (C/EBP)-β binding sites within the putative proximal promoters of the *Klk1* gene, hinting that IL-17 may regulate *Klk1* expression in a C/EBPβ-dependent manner. Indeed, C/EBPβ^-/-^ mice are susceptible to systemic candidiasis [48,49]. Since TNFα is required for protection against disseminated candidiasis [50], further studies should focus on *Klk* gene expression in the kidney of TNFα-deficient mice.

Klk1 cleaves kininogens to form bradykinin, a process known as the bradykinin-dependent pathway [20]. In addition, Klk1-mediated activation of PAR4 induces cytokine production and prevents apoptosis in RTEC, known as the bradykinin-independent pathway [37]. Mice deficient in specific PARs or treated with PAR antagonists exhibited compromised renal inflammatory changes [51,52]. Nevertheless, the contribution of PARs in IL-17-Klk1-mediated renal protection is unknown. Moreover, the relative contributions of Bdkrb1 and Bdkrb2 in renal defense against candidiasis need to be determined, which is possible with specific knockout mouse strains.

Our data show that bradykinin treatment improved renal function as early as 7 days p.i. Interestingly, serum creatinine and BUN levels did not correlate with the survival benefit at late time points, the basis for which is unclear. Late mortality despite improved renal function likely indicates side effects of bradykinin in mice. Although bradykinin has been implicated in causing angioedema [37], mice treated with bradykinin did not show any signs of angioedema following fungal infection (Fig 4C). Additionally, bradykinin at the dose levels used in this study did not seem to exert any long term consequences on blood pressure. Therefore, these studies argue against the likelihood of bradykinin toxicity and point towards alternative possibilities to explain the lack of correlation between kidney function and survival benefit. First, extremely short half-life of bradykinin (approx. 30 sec) and internalization of bradykinin receptors may lead to bradykinin unresponsiveness at the late time points. Second, daily administration of bradykinin may lead to the activation of a negative feedback regulatory mechanisms leading to increased degradation of bradykinin by Angiotensin converting enzymes. Finally, bradykinin has been shown to modulate glomerular function by regulating podocyte permeability [53], an effect independent of its renal tissue protective function. Given the potential side effects in bradykinin therapy, future studies investigating these questions could help better understand the means to harness the beneficial impact of IL-17-KKS axis without compromising the safety in treatment against disseminated candidiasis.

An Ab targeting IL-17 (secukinumab) was approved in 2016 to treat moderate-severe plaque psoriasis [54]. Abs against IL-17/IL-17RA are in clinical trials for other autoimmune conditions [55]. One obvious concern with anti-IL-17 therapy is compromising IL-17-driven antifungal immunity. Although systemic *C*. *albicans* infection has not been reported thus far with secukinumab, patients on this medication may have a higher risk of developing disseminated candidiasis in the face of predisposing factors, such as an indwelling catheter, abdominal surgery or long-term antibiotic use. Our data show that activation of the KKS pathway restored protection in IL-17RA^-/-^ mice. Thus, drugs targeting the IL-17-KKS axis may be considered to treat or prevent disseminated *C*. *albicans* infection in patients receiving anti-IL-17 therapy.

Amphotericin B, azoles and echinocandins are used to treat systemic *C*. *albicans* infections, but there are concerns due to drug-resistance and toxicity. Here, we show proof-of-concept that combination therapy with bradykinin and low-dose FLC is effective in treating candidiasis in mice. Notably, the survival rate of mice treated with the combination therapy was similar to the survival rate in mice given four times the FLC dose. This approach may be especially valuable for anti-fungal drugs such as amphotericin B due its intrinsic renal toxicity. Future studies will test the efficacy of combination therapy with amphotericin B and bradykinin, once we have access to oral or injectable form of amphotericin B suitable for administration in mice. In addition, the efficacy of ACE inhibitors, known to increase the levels of bradykinin and are routinely used to treat patients with obstructive nephropathy, need further evaluation in pre-clinical animal models of disseminated candidiasis. Overall, our data show that defining the IL-17 anti-fungal pathway has highlighted a potentially translatable and testable approach to treating systemic candidiasis. Additionally, the novel convergence between IL-17 and the KKS pathways in renal defense against fungal infection represents a major advance in our understanding of IL-17 signaling in the kidney inflammation.

## Material and Methods

### Ethics Statement

Wild type (WT) C57BL/6J mice were purchased from The Jackson Laboratory (Bar Habor, ME). IL-17RA^-/-^ mice were kindly provided by Amgen (San Francisco, CA) and bred in-house. All mice were housed under specific pathogen-free conditions, and age-matched male mice were used for all experiments. Animal protocols were approved by the University of Pittsburgh IACUC (Protocol # 14094427), and adhered to the guidelines in the Guide for the Care and Use of Laboratory Animals of the National Institute of Health.

### *C*. *albicans* culture and disseminated infection

*C*. *albicans* strains CAF2-1 or SC5314 were used as indicated. *C*. *albicans* was grown in YPD at 30°C for 18–24 h. Mice were injected via the tail vein with PBS (sham-infected) or 1x10^5^ cfu (unless otherwise indicated) *C*. *albicans* yeast cells resuspended in PBS. Mice were weighed and monitored daily. Mice were sacrificed if they showed >20% weight loss or signs of severe pain or distress. Mice were evaluated for survival over a period of 14 days. At sacrifice on days 2, 3 and 7 p.i., as indicated, kidneys were weighed and homogenized in sterile PBS using a GentleMACS (Miltenyi Biotec, Cambridge MA). Serial dilutions of organ homogenates were plated on YPD agar with antibiotics, and fungal burden represented as colony forming units (cfu) per gram of tissue.

### Measurement of serum creatinine and blood urea nitrogen

Serum was collected by retro-orbital bleeding at day 7 p.i. Creatinine and blood urea nitrogen levels were assessed using the QuantiChrom Creatinine Assay kit (BioAssay Systems, Hayward CA) and MaxDiscovery Blood Urea Nitrogen Enzymatic kit (Bioo Scientific Corp., Austin TX), respectively.

### Measurement of vascular permeability

Vascular permeability in mice was assessed as described before [56]. Briefly, 100 μl sterile solution of Evans Blue dye (30mg/kg) (Sigma Aldrich., St Louis, MO) in PBS was injected intravenously. The stain was allowed to circulate for 30 min. After 30 min, mice were sacrificed and the hind feet were removed, blotted dry and weighed. The Evans blue was extracted from the feet with 1 ml of formamide overnight at 55°C and measured spectrophotometrically at 600 nm. Evans Blue stain was quantified according to a standard curve. The results are presented as ng of Evans Blue stain/mg of tissue.

### *In vitro* stimulation of primary renal tubular epithelial cells and macrophages

Primary renal tubular epithelial cells (RTEC) from C57BL/6J mice (Cell Biologics, Chicago, IL) were cultured as per manufacturer’s instructions. RTEC (1x10^6^ cells/well) were treated with IL-17A (50 or 200 ng/ml) or TNFα (5 ng/ml) or IL-17C (50 or 200 ng/ml) or IL-17F (50 or 200 ng/ml) and IL-17 and TNFα in combination for 24 h. Recombinant murine IL-17A, IL-17C, IL-17F and TNFα were purchased from Peprotech (Rocky Hill, NJ).

Bone marrow derived macrophages (BMDM) from C57BL/6J mice were cultured for 7 days in the presence of L929 supernatants. BMDM and RTEC (1x10^6^ cells/well) were treated with bradykinin (R&D Biosystems, Minneapolis MN) or left untreated for 24 h. LPS (Sigma Aldrich, St Louis, MO) was used as positive control. Supernatants were subjected to analyses using commercially available IL-6, IL-1β and TNFα ELISA kits (Ebiosciences, Dallas TX. Nitrite concentrations were measured by tri-iodide based reductive chemiluminescence as previously described [57]. Briefly, samples were injected into tri-iodine to reduce nitrite to NO gas that was detected by a Nitric Oxide Analyzer (Sievers, GE).

### *In vitro* fungal killing assay

Neutrophils isolated from bone marrow using Neutrophil Isolation Kit (Miltenyi Biotech, San Diego, CA) were plated at 1×10^5^ cells/well. Non-opsonized *C*. *albicans* was added to neutrophils at 0.5 × 10^5^ yeast cells/well (ratio of 2:1). If indicated, bradykinin was added to the wells. Cultures were incubated with unopsonized *C*. *albicans* for 3 h and lysed in cold double-distilled H_2_O. Killing was assessed by cfu counts in triplicate. The results are reported as percentage killing of *C*. *albicans* which is calculated as 1-[cfu of treatment group/cfu of control group] X 100. The same protocol was followed for bone marrow derived macrophages which were cultured with L929 supernatants supplemented media for 7 days prior to carrying out the killing assay.

### Gene expression profiling and quantitative real–time PCR analysis

At sacrifice, kidneys were stored at -80°C. Total RNA was extracted from the homogenized kidney tissue with the RNeasy Micro Kit (Qiagen, Valencia CA) and submitted to Genomics Research Core at University of Pittsburgh. Gene expression analysis was performed using Mouse WG6 Gene Expression Bead Chip (Illumina). All test, normalization and transformation analyses were performed using caGEDA, a feely available informatics tool. The data sets were analyzed for differentially expressed genes. Efficiency analysis was performed by Random Resampling Validation using a Naïve Bayes Classifier and PACE analysis. The cluster analysis was performed by Unweighted Pair Group Method with Arithmetic Mean and similarity measure was determined by Euclidean distance. The raw and normalized microarray data have been submitted to the Gene Expression Omnibus (GEO), and study ID is GSE88800 at: https://www.ncbi.nlm.nih.gov/geo/query/acc.cgi?acc=GSE88800. For real time PCR analysis, complementary DNA was synthesized with SuperScript III First-Strand (Invitrogen, Carlsbad CA). Gene expression was determined by qPCR with PerfeCTa SYBR Green FastMix ROX (Quanta BioSciences, Gaithersburgh MD) on a 7300 Real-Time PCR System (Applied Biosystems, Carlsbad CA). Primers were obtained from Quantitect (Qiagen, Valencia CA). The expression of each gene was normalized to *Gapdh*.

### Flow cytometry

At sacrifice on day 7 p.i., kidneys were harvested following perfusion with PBS. Briefly, kidney homogenates were digested in PBS with 1 mg/ml collagenase type I (Worthington, Lakewood NJ) for 30 m at 37°C. Cells were stained with the following antibodies: CD45 (BioLegend; clone 30-F11), Ly6G (BD Biosciences; clone IA8), F/480 (eBiosciences; clone BM8). For detection of apoptotic cells and cleaved Caspase-3 positive cells, cells were stained with Annexin V (BD Pharmingen) and CaspGLOW Fluroscein active Caspase-3 staining kit (eBioSciences), respectively as per manufacturer’s protocol. Samples were acquired and sorted on a Fortessa and FACS ARIA II, respectively (BD Biosciences, San Jose CA) and analyzed with FlowJo (Tree Star, Ashland OR).

### Histology and Immunohistochemistry

Kidneys were fixed in formalin, dehydrated and paraffin embedded. Serial kidney sections were stained with H&E, Periodic-acid Schiff or Masson Trichrome stains for morphological analysis and determination of kidney injury.

Immunohistochemistry staining was done on formalin-fixed, paraffin embedded sections. Sections were rehydrated and antigen retrieval was performed with heated citrate. Primary antibodies against the following proteins were used: Klk1 (LifeSpan Biosciences, Seattle WA), NGAL (Santa Cruz Biotechnology, Dallas TX) and cleaved caspase-3 (Cell Signaling, Danvers MA). Secondary antibodies used were horseradish peroxidase coupled antibodies (Jackson ImmunoResearch, West Grove, PA). To detect apoptotic cells TUNEL staining was done on frozen kidney sections using the TUNEL apoptosis detection kit according to manufacturer’s protocol (Millipore, Temecula CA). The number of TUNEL^+^ cells was counted in 15 randomly selected high powered fields (400X) per slide. All images were obtained with EVOS FL Auto microscope (Life Technologies CA).

### Western blots

Kidneys were homogenized in RIPA buffer. Concentration of protein was quantified by the BCA quantitation assay (Thermo Scientific, Pittsburgh PA). Equal amounts of sample were subjected to electrophoresis and transferred to PVDF membranes (Millipore, Billerica MA). After blocking with 5% milk in TBS, the blots were incubated with anti-mouse Klk1 (LifeSpan Biosciences, Seattle WA), anti-mouse NGAL (R&D Biosystems, Minneapolis MN), anti-mouse cleaved Caspase-3 (Cell Signaling, Danvers MA) or anti-mouse beta-actin (Abcam, Cambridge, MA) overnight in 4°C. The blots were then washed and incubated for 1 hour at room temperature with individual secondary antibodies. Bands were detected using an enhanced chemiluminescence detection system (ThermoScientific, Pittsburgh PA) and developed with a FluorChem E imager (ProteinSimple, San Jose CA). Band corresponding to proteins of interest were analyzed by ImageJ software.

### Adenoviruses

Adenoviruses expressing IL-17A (Ad-IL-17) and control vector (Ad-ctrl) were kindly provided by Dr. J. Kolls (U. Pittsburgh). Ad-Klk1 and corresponding Ad-ctrl vector were from Applied Biological Materials Inc. (Richmond, British Columbia, Canada). Mice were injected via the tail vein with 1x10^9^ pfu virus 72 h prior to induction of disseminated candidiasis.

### Bradykinin receptor agonists and antagonists

Mice were injected with bradykinin **(**300 nmol/kg/day) (R&D Systems, Minneapolis MN) in a 200 μl volume i.p. Mice received i.p. injection of a combination of Bdkrb1 (R-715: 1 mg/kg/day) and Bdkrb2 (HOE-140: 1 mg/kg/day) antagonists (R&D Systems, Minneapolis MN). Untreated mice received equal volume of PBS.

### Fluconazole treatment

*C*. *albicans* infected mice were treated with Fluconazole (FLC) (Diflucan: obtained from University of Pittsburgh Medical Center, Pittsburgh PA) as described before with minor modifications [58]. Briefly, mice were treated with 5, 10, 20 and 40 mg/kg body weight FLC by oral gavage at 2 h and 26 h post infection. Untreated mice received equal volume of PBS.

### Statistics

Data were analyzed by Kaplan-Meier, ANOVA, Mann-Whitney or unpaired Student's *t* test using GraphPad Prism (La Jolla, CA). *P* values <0.05 were considered significant. All experiments were performed a minimum of twice to ensure reproducibility.

## Supporting Information

S1 Fig Klk genes were not induced in RTEC following IL-17C and IL-17F stimulation. **(A)** WT and IL-17RA^-/-^ mice (n = 5) were subjected to systemic *C*. *albicans* infection. At 48 h p.i., transcript expression of *Cxcl1*, *Cxcl2* and *Lcn2* were quantified by qPCR. Each dot represents individual mice and bars indicate mean. **(B)** WT mice (n = 6) were either infected with 1x10^5^ or 1x10^6^ cfu *C*. *albicans*. After 48 h, kidneys were evaluated for mRNA expression of Klk1. Each dot represents individual mice and bars indicate mean. Data are pooled from two independent experiments for **(A)** and **(B)**. **(C)** RTEC from WT mice were treated with IL-17C (50ng/ml and 200ng/ml), TNF-α (5ng/ml) or IL-17C (200ng/ml) + TNF-α (5ng/ml) (upper panel) or IL-17F (50ng/ml and 200ng/ml), TNF-α (5ng/ml) or IL-17F (200ng/ml) + TNF-α (5ng/ml) (lower panel) for 24 h. Cells were evaluated for mRNA expression of *Klk1* and *Klk1b27* by qPCR. Data are representative of 3 independent experiments. Bars represent mean ± S.D. ns, not significant.
(TIF)

S2 Fig Increased serum IL-17 and IL-17-responsive gene expression in the kidney of Ad-IL-17 injected mice. WT mice (n = 4) were infected with adenovirus expressing IL-17 (Ad-IL-17) or control vector (Ad-ctrl). Six days post-infection, mice were evaluated for (**A**) serum IL-17 level (**B**) IL-17-responsive gene expression in the kidney by qPCR (**C**) Serial kidney sections were stained for H&E to evaluate renal inflammatory changes. Bars represent mean ± S.D. **(D)** WT (n = 8) mice were either injected with Ad-Klk1 or Ad-ctrl vector 72 h prior to systemic *C*. *albicans* infection. After 72 h, mice were assessed for inflammatory gene expression in the kidney. Each dot represents individual mice and bars indicate mean. Data are pooled from two independent experiments. *P* <0.05 (*), <0.0001 (****). ns, not significant.
(TIF)

S3 Fig Administration of bradykinin caused brief hypotensive response followed by recovery and no change in kidney infiltrating leukocytes. **(A)** Mean arterial BP was measured by direct arterial cannulation method in mice (n = 2) every 2 min (Baseline) and every 1 min after i.p. injection of bradykinin (300 nmol/kg/day). PBS was injected as control. **(B)** WT mice were treated daily with bradykinin (300 nmol/kg/day**)** starting day -1 (relative to infection). At day 0, mice were subjected to systemic candidiasis. As a negative control, sham- infected WT mice were treated with bradykinin only. At day 7 p.i. (n = 4–6), kidney infiltrating neutrophils (Gr1^+^) and macrophages (F4/80^+^) (gated on CD45^+^) cells were evaluated by flow cytometry. The numbers in the FACS plot indicate percentage of cells. The FACS plot is representative of 4–6 mice/group from two independent experiments.
(TIF)

S4 Fig Renal fibrotic changes and tissue growth factors expression are comparable between bradykinin treated and untreated mice. WT mice (n = 6–8) were treated daily with bradykinin (300 nmol/kg/day**)** starting day -1 (relative to infection). At day 0, mice were subjected to systemic candidiasis. As a negative control, sham- infected WT mice were treated with bradykinin only (n = 4). (**A**) At day 7 post infection, serial kidney sections were subjected to Masson-trichome staining to determine fibrotic changes. Photomicrographs are representative of two individual experiments. Original magnification: 100X. **(B)** Kidneys were evaluated for expression of *Ctgf*, *Hgf*, *Mmp2* and *Mmp9* by qPCR. Each dot represents an individual mouse and the bars indicate mean for each group. Data are pooled from three independent experiments. *ns*, not significant.
(TIF)

S5 Fig Determination of minimally effective dose of fluconazole in disseminated candidiasis. WT mice (n = 8) were either infected with *C*. *albicans* or left sham-infected. Two hours p.i., mice were either treated with the first dose of FLC by oral gavage at 5, 10, 20 and 40 mg /kg body weight or left untreated. A second dose of FLC was administered 24 h p.i. Sham-infected mice (n = 4) were treated with 5, 10, 20 and 40 mg /kg FLC. **(A)** On day 4 p.i., fungal load in the kidney was assessed. **(B)** Survival was assessed over 14 d. Each dot represent individual mice and bars indicate mean for each group. Data are pooled from two independent experiments. p<0.05 (*), p>0.01 (**), p>0.0001 (****). ns, not significant.
(TIF)

S6 Fig The IL-17-KKS-axis confers renal protection against disseminated candidiasis. In response to disseminated candidiasis, kidney infiltrating innate and adaptive IL-17-producing cells is the major source of IL-17. IL-17 in turn binds its receptor (IL-17RA/RC) on kidney-resident target cells, activating downstream signaling events leading to expression of IL-17-responsive cytokines, chemokines and AMP genes. Innate effectors (neutrophils, macrophages) recruited in response to IL-17-induced signals facilitate fungal clearance. IL-17 also induces expression of kallikreins in target cells. Kallikreins cleave kininogens to form bradykinin. Activation of bradykinin receptors (Bdkrb) on renal cells prevents apoptosis and controls of tissue damage.
(TIF)
