# Supplementary figures and images for: The Kallikrein-Kinin System: A Novel Mediator of IL-17-Driven Anti-Candida Immunity in the Kidney
Source: PLoS Pathog. 2016 Nov 4;12(11):e1005952. doi: 10.1371/journal.ppat.1005952 (PMC5096720; doi:10.1371/journal.ppat.1005952)

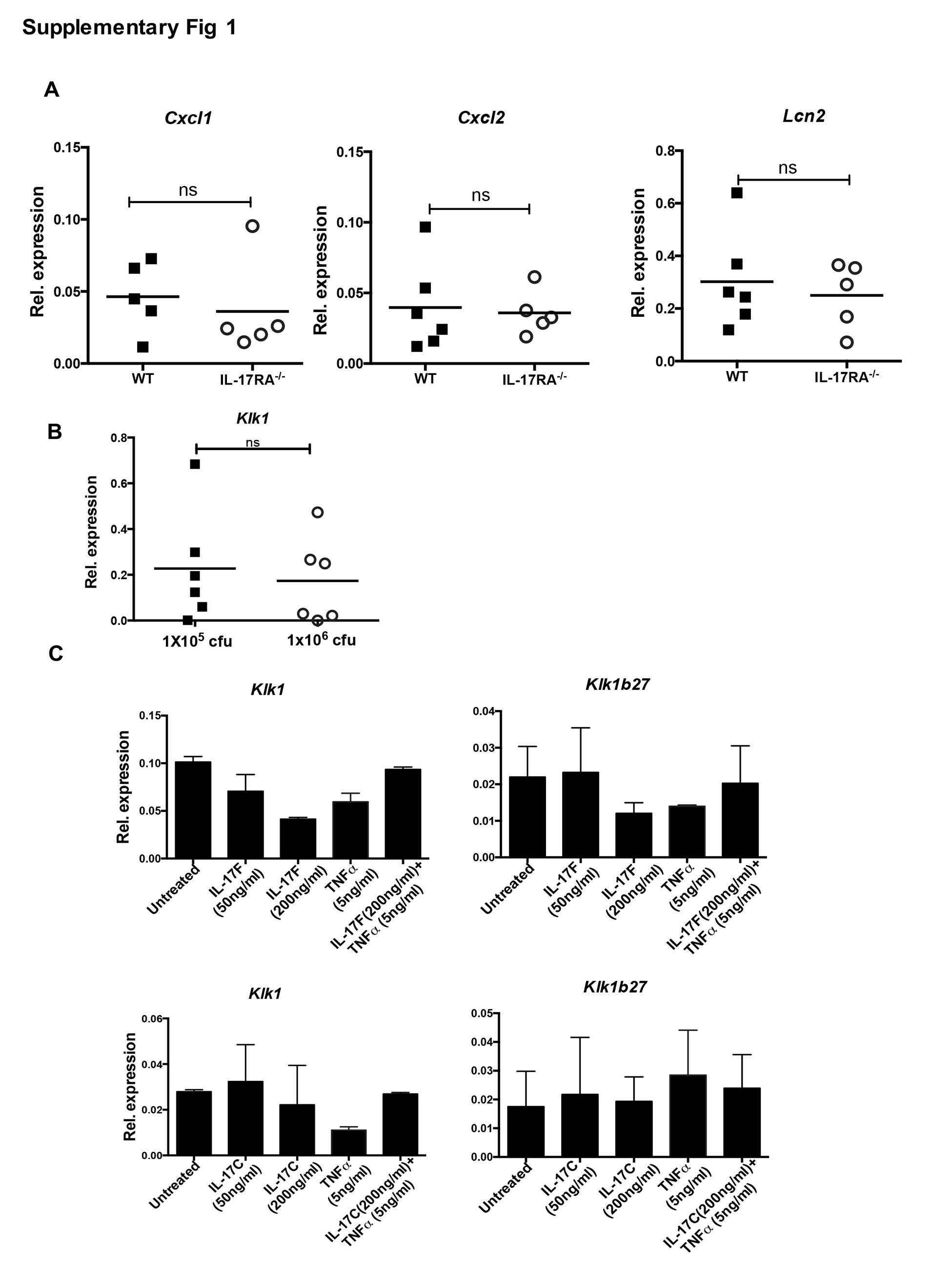

Supplement: S1 Fig — (A) WT and IL-17RA-/- mice (n = 5) were subjected to systemic C. albicans infection. At 48 h p.i., transcript expression of Cxcl1, Cxcl2 and Lcn2 were quantified by qPCR. Each dot represents individual mice and bars indicate mean. (B) WT mice (n = 6) were either infected with 1x10^5^ or 1x10^6^ cfu C. albicans. After 48 h, kidneys were evaluated for mRNA expression of Klk1. Each dot represents individual mice and bars indicate mean. Data are pooled from two independent experiments for (A) and (B). (C) RTEC from WT mice were treated with IL-17C (50ng/ml and 200ng/ml), TNF-α (5ng/ml) or IL-17C (200ng/ml) + TNF-α (5ng/ml) (upper panel) or IL-17F (50ng/ml and 200ng/ml), TNF-α (5ng/ml) or IL-17F (200ng/ml) + TNF-α (5ng/ml) (lower panel) for 24 h. Cells were evaluated for mRNA expression of Klk1 and Klk1b27 by qPCR. Data are representative of 3 independent experiments. Bars represent mean ± S.D. ns, not significant. (TIF) [file ppat.1005952.s001.tif]

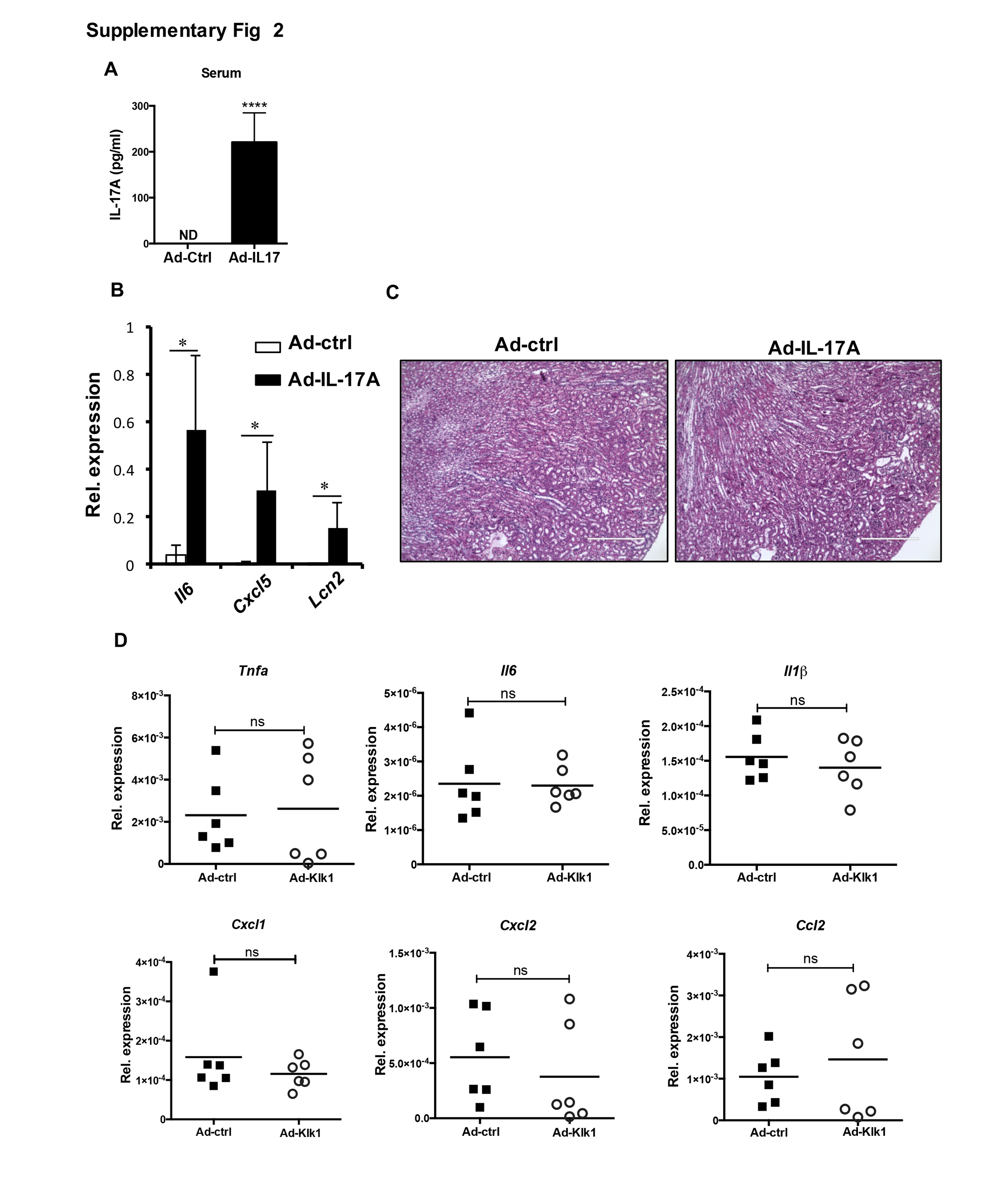

Supplement: S2 Fig — WT mice (n = 4) were infected with adenovirus expressing IL-17 (Ad-IL-17) or control vector (Ad-ctrl). Six days post-infection, mice were evaluated for (A) serum IL-17 level (B) IL-17-responsive gene expression in the kidney by qPCR (C) Serial kidney sections were stained for H&E to evaluate renal inflammatory changes. Bars represent mean ± S.D. (D) WT (n = 8) mice were either injected with Ad-Klk1 or Ad-ctrl vector 72 h prior to systemic C. albicans infection. After 72 h, mice were assessed for inflammatory gene expression in the kidney. Each dot represents individual mice and bars indicate mean. Data are pooled from two independent experiments. P <0.05 (*), <0.0001 (****). ns, not significant. (TIF) [file ppat.1005952.s002.tif]

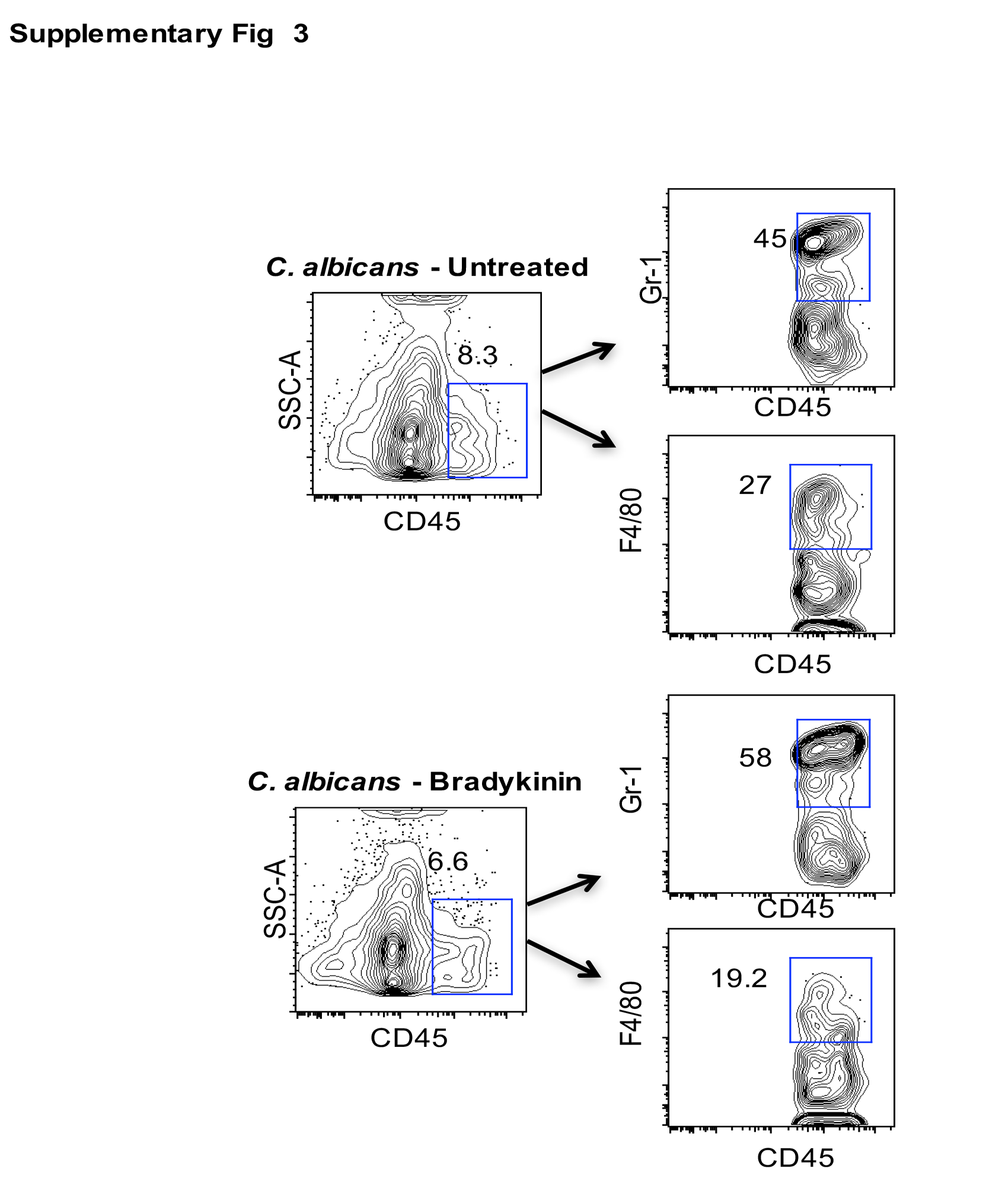

Supplement: S3 Fig — (A) Mean arterial BP was measured by direct arterial cannulation method in mice (n = 2) every 2 min (Baseline) and every 1 min after i.p. injection of bradykinin (300 nmol/kg/day). PBS was injected as control. (B) WT mice were treated daily with bradykinin (300 nmol/kg/day) starting day -1 (relative to infection). At day 0, mice were subjected to systemic candidiasis. As a negative control, sham- infected WT mice were treated with bradykinin only. At day 7 p.i. (n = 4–6), kidney infiltrating neutrophils (Gr1+) and macrophages (F4/80+) (gated on CD45+) cells were evaluated by flow cytometry. The numbers in the FACS plot indicate percentage of cells. The FACS plot is representative of 4–6 mice/group from two independent experiments. (TIF) [file ppat.1005952.s003.tif]

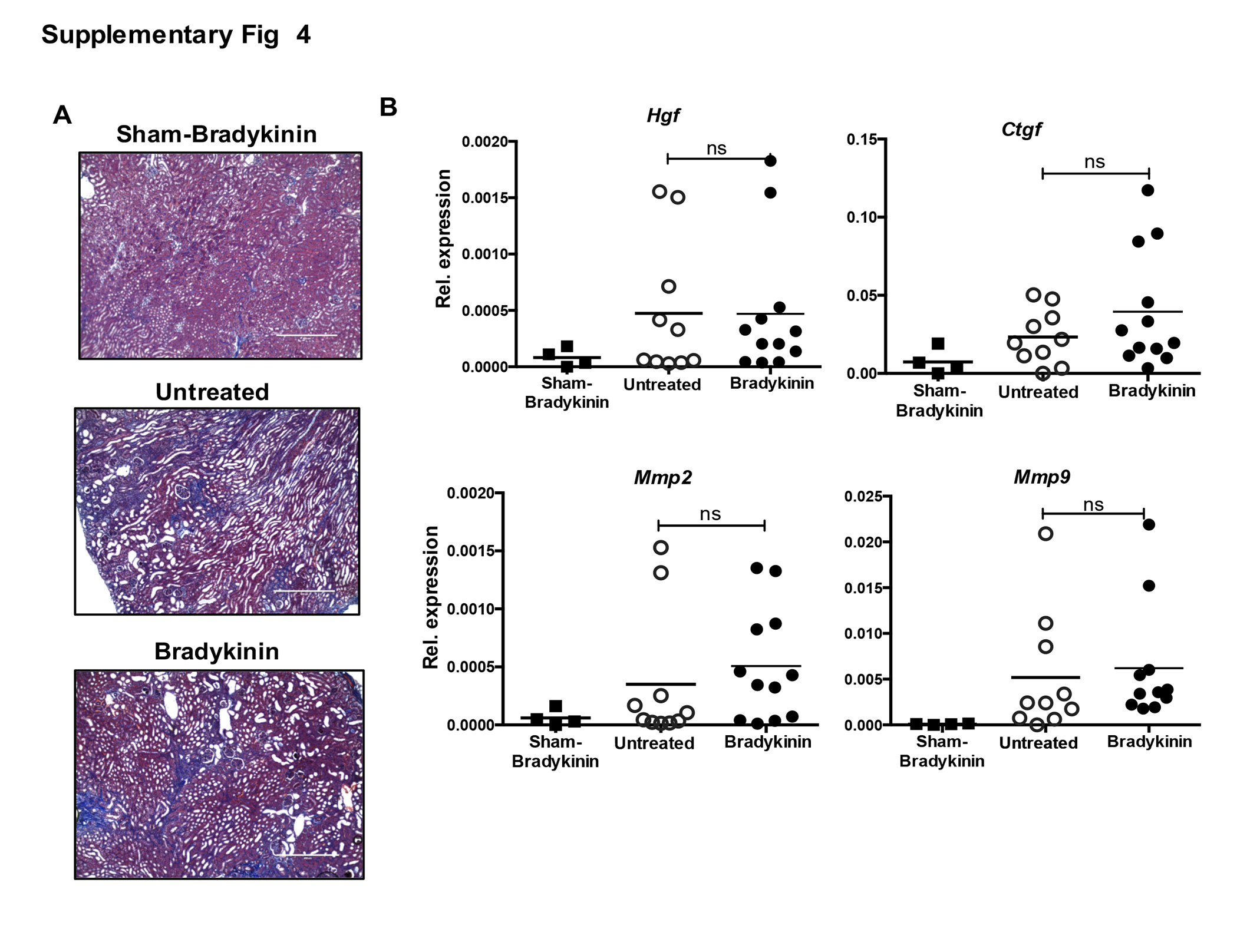

Supplement: S4 Fig — WT mice (n = 6–8) were treated daily with bradykinin (300 nmol/kg/day) starting day -1 (relative to infection). At day 0, mice were subjected to systemic candidiasis. As a negative control, sham- infected WT mice were treated with bradykinin only (n = 4). (A) At day 7 post infection, serial kidney sections were subjected to Masson-trichome staining to determine fibrotic changes. Photomicrographs are representative of two individual experiments. Original magnification: 100X. (B) Kidneys were evaluated for expression of Ctgf, Hgf, Mmp2 and Mmp9 by qPCR. Each dot represents an individual mouse and the bars indicate mean for each group. Data are pooled from three independent experiments. ns, not significant. (TIF) [file ppat.1005952.s004.tif]

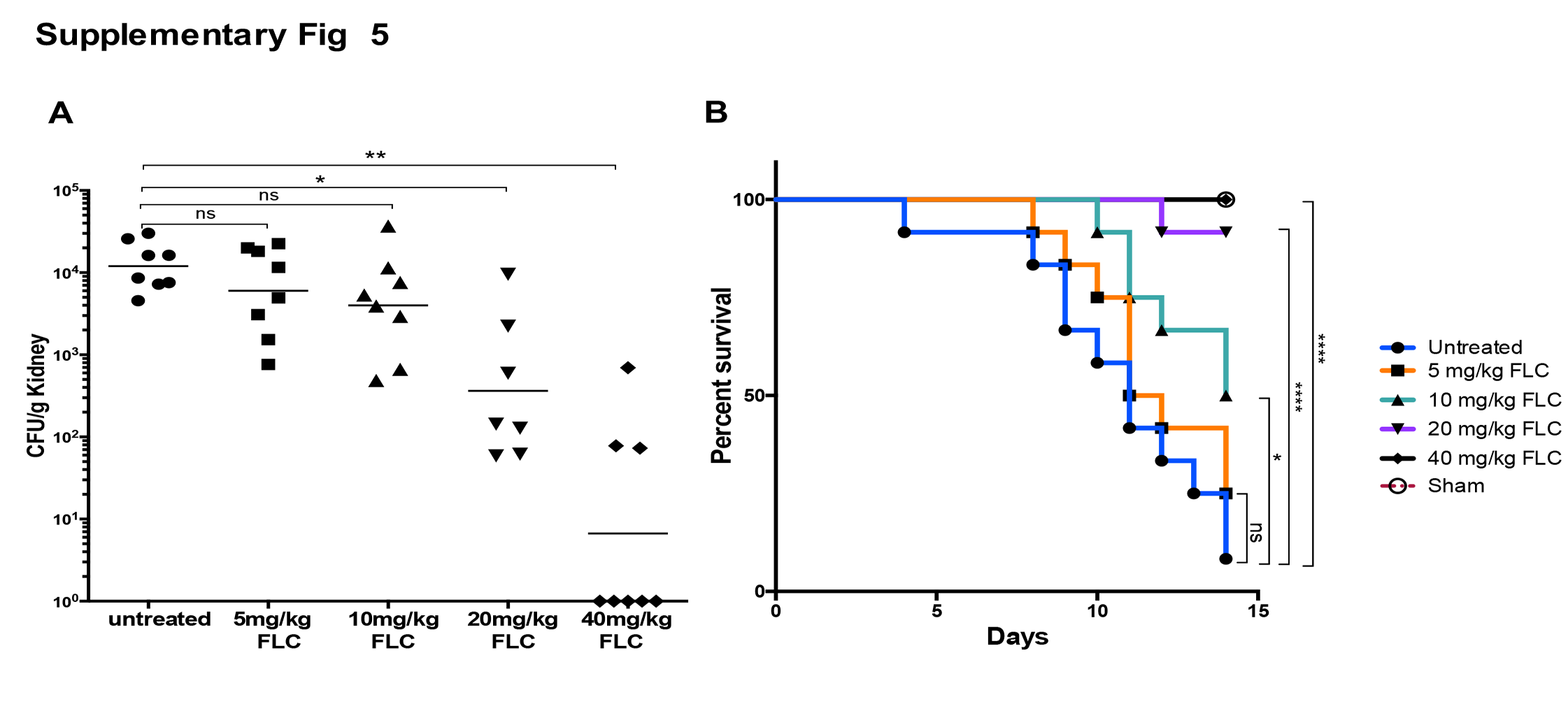

Supplement: S5 Fig — WT mice (n = 8) were either infected with C. albicans or left sham-infected. Two hours p.i., mice were either treated with the first dose of FLC by oral gavage at 5, 10, 20 and 40 mg /kg body weight or left untreated. A second dose of FLC was administered 24 h p.i. Sham-infected mice (n = 4) were treated with 5, 10, 20 and 40 mg /kg FLC. (A) On day 4 p.i., fungal load in the kidney was assessed. (B) Survival was assessed over 14 d. Each dot represent individual mice and bars indicate mean for each group. Data are pooled from two independent experiments. p<0.05 (*), p>0.01 (**), p>0.0001 (****). ns, not significant. (TIF) [file ppat.1005952.s005.tif]

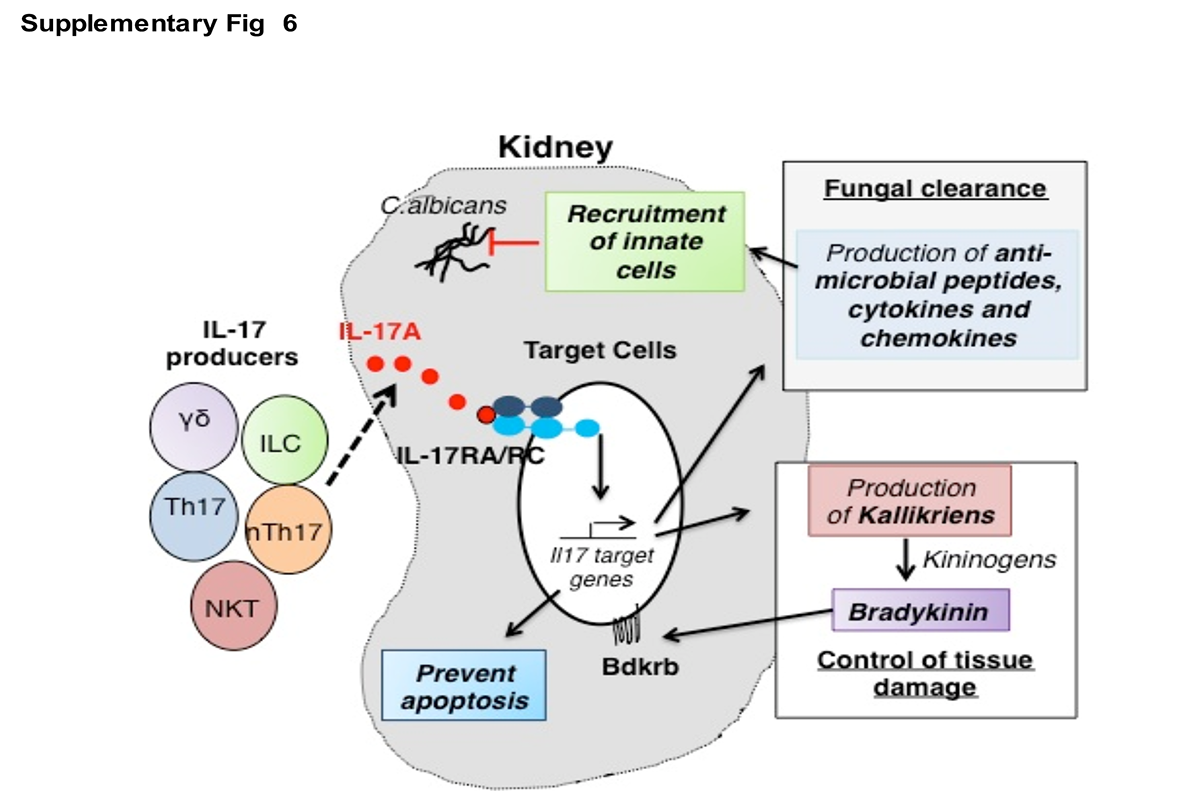

Supplement: S6 Fig — In response to disseminated candidiasis, kidney infiltrating innate and adaptive IL-17-producing cells is the major source of IL-17. IL-17 in turn binds its receptor (IL-17RA/RC) on kidney-resident target cells, activating downstream signaling events leading to expression of IL-17-responsive cytokines, chemokines and AMP genes. Innate effectors (neutrophils, macrophages) recruited in response to IL-17-induced signals facilitate fungal clearance. IL-17 also induces expression of kallikreins in target cells. Kallikreins cleave kininogens to form bradykinin. Activation of bradykinin receptors (Bdkrb) on renal cells prevents apoptosis and controls of tissue damage. (TIF) [file ppat.1005952.s006.tif]
